# Supplementary material for: Neurocomputational mechanisms of affected beliefs
Source: Commun Biol. 2022 Nov 14;5:1241. doi: 10.1038/s42003-022-04165-3 (PMC9663730; doi:10.1038/s42003-022-04165-3)
Supplement: Supplementary file 2 — Supplementary Information [file 42003_2022_4165_MOESM2_ESM.pdf]

Supplementary Information

Title: Neurocomputational mechanisms of affected beliefs

Authors: Laura Müller-Pinzler[1]*, Nora Czekalla[1], Annalina V. Mayer[1], Alexander Schröder[1], David S. Stolz[1], Frieder M. Paulus[1], Sören Krach[1]

Email: Laura Müller-Pinzler* laura.muellerpinzler@uni-luebeck.de

Nora Czekalla

Annalina V Mayer

Alexander Schröder

David S Stolz

Frieder M Paulus

Sören Krach

Affiliations:

1: Department of Psychiatry and Psychotherapy, Social Neuroscience Lab, University of Lübeck, Ratzeburger Allee 160, D-23538 Lübeck, Germany

***Corresponding Author:**

Dr. Laura Müller-Pinzler

Department of Psychiatry and Psychotherapy, Social Neuroscience Lab

University of Lübeck, Ratzeburger Allee 160, D-23538 Lübeck, Germany

Phone: +49 45131017529

Supplementary Figures

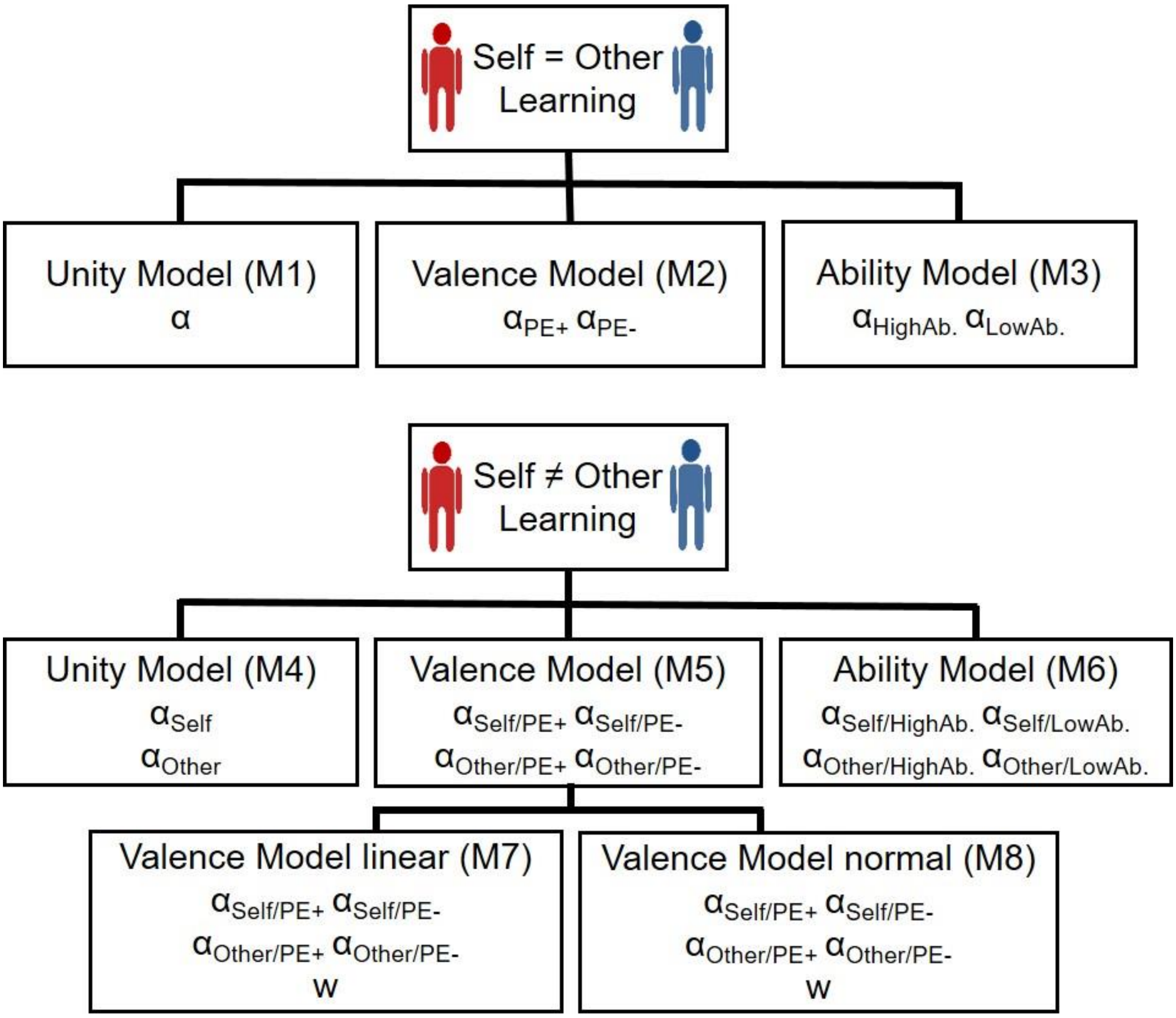

Supplementary Figure 1. Structure of the model space. Two factors were distinguished that impact learning rates (α): the agent (self vs other) and the impact (no impact: Unity Model) of prediction error valence (Valence Model) or the ability condition (Ability Model). The Valence Model, winning model in previous studies [1], was extended by a decay factor (w) for the learning rates towards the ends of the feedback scale with a linear decrease (Valence Model linear) or a decrease following the relative probability density of the normal distribution (Valence Model normal; for more details see **Methods**).

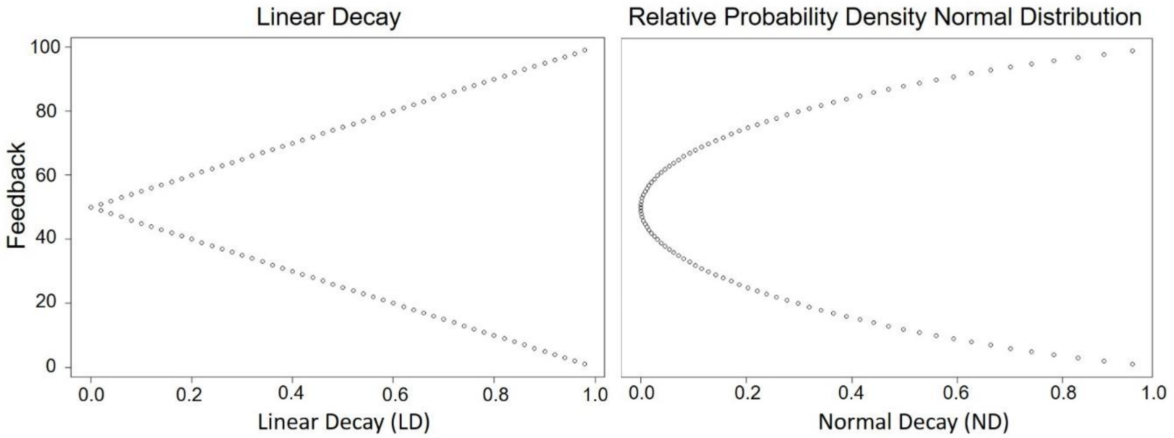

Supplementary Figure 2. Depiction of the linear decay (left) and the decay following the relative probability density of the normal distribution (right) for the different feedback values. The values depicted here were introduced in the learning models and weighted by a weighting factor as described in the **Methods** section.

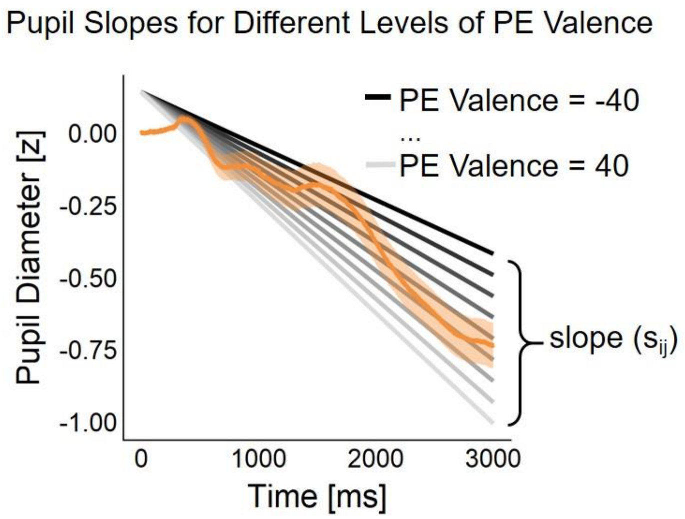

Supplementary Figure 3. Average pupil trace and pupil slopes for different levels of PE Valence. More negative PEs are associated with greater pupil slopes. The average pupil diameter trace during feedback is depicted in orange, the shaded area represents +/- one standard error. Pupil slopes for the different levels of PEs (from black = negative to grey = positive) were predicted by the multi-level model containing PE valence and PE surprise as predictors, as described in the **Methods** section.

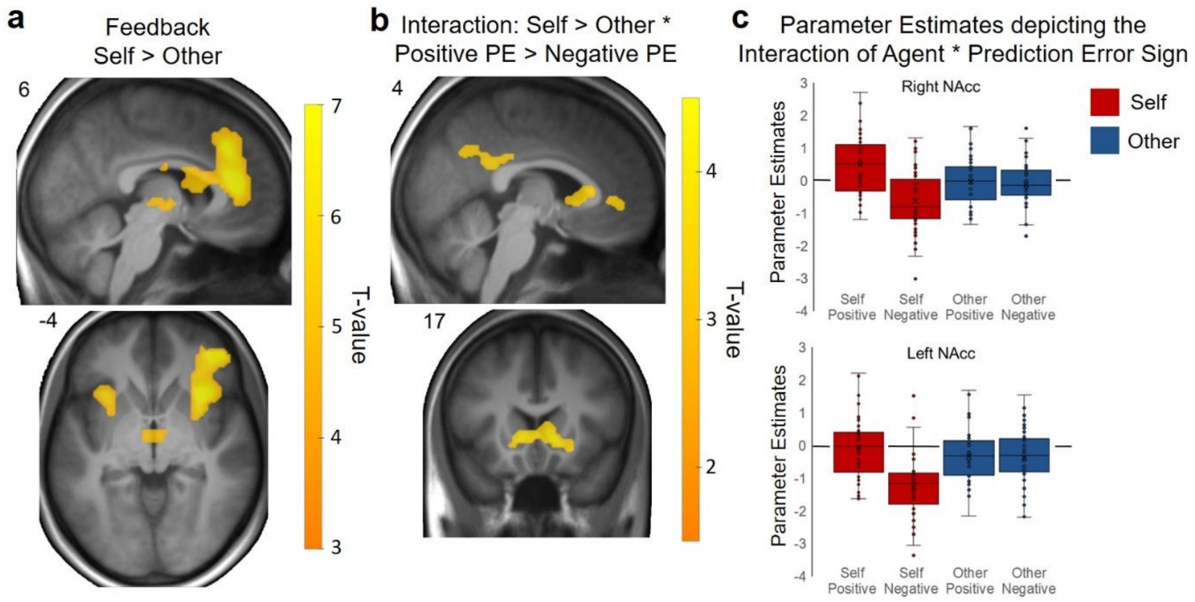

Supplementary Figure 4. Neural activations associated with feedback processing. a) Self-related feedback vs. other-related feedback was associated with an increased activation of the mPFC/ ACC, bilateral anterior insula and thalamus, among other regions ($p < .05$, FWE corrected at peak level for the whole brain). **b)** The interaction of Agent and Predition Error Sign (([Self positive PE > Self negative PE] > [Other positive PE > Other negative PE]) resulted in activation of the angular gyrus, the bilateral NAcc/VS, the precuneus/ posterior cingulate cortex, and precentral gyrus (cluster-wise FWE corrected with $p < .05$ at a cluster forming threshold of $p < .001$ for displaying purposes). **c)** Parameter estimates correspond to the BOLD response to positive and negative PEs in bilateral NAcc/ VS [left: x, y, z: -9 20 -1; right: x, y, z: 12 20 -1]. The plot shows that positive relative to negative PEs increased the activity in NAcc/ VS only when learning about the own performance, but not when observing others. Colored bars indicate the first and third quartile of the data, the line marks the median, the cross marks the mean. Whiskers extend from the upper (lower) box borders to the largest (smallest) data point at most 1.5 times the interquartile range above (below) the respective border. Data with more extreme values than this are displayed as individual points.

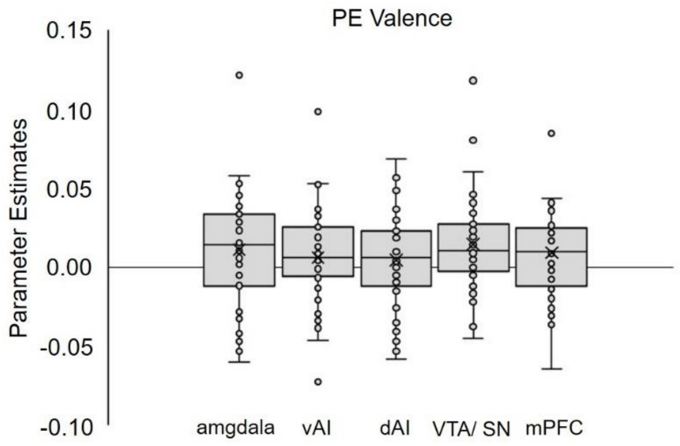

Supplementary Figure 5. Parameter estimates for the PE Valence effect in our predefined ROIs (amygdala, vAI, dAI, VTA/SN, and mPFC). Parameter estimates were derived from all voxels within each ROI and averaged across all voxels. Left and right amygdala, vAI, and dAI were combined bilaterally for displaying purposes. Dots show the data for individual subjects, grey bars show the first and third quartile, the cross marks the mean and the line the median. Whiskers extend from the upper (lower) box borders to the largest (smallest) data point at most 1.5 times the interquartile range above (below) the respective border. Data with more extreme values than this are displayed as individual points. All regions show variance between subjects in such a way that some individuals have positive values and other negative values indicating stronger activity scaling with more positive or more negative PEs, respectively.

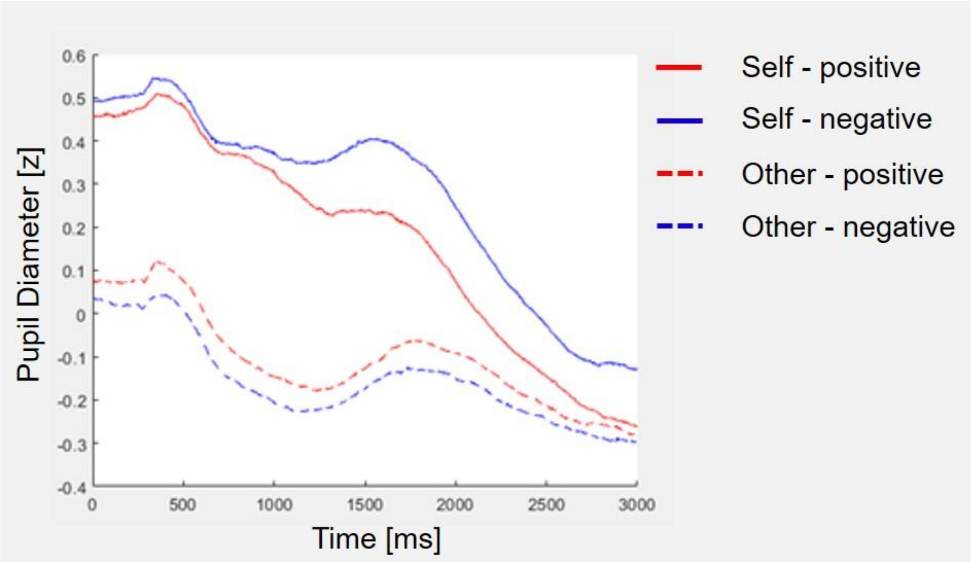

Supplementary Figure 6. Pupil diameter traces for the feedback phase (3 secs) for all four feedback conditions uncorrected for offsets at feedback start. The bold lines show pupil traces for Self and the dashed lines for Other. Red lines show pupil traces for feedback with positive PEs and blue lines for negative PEs.

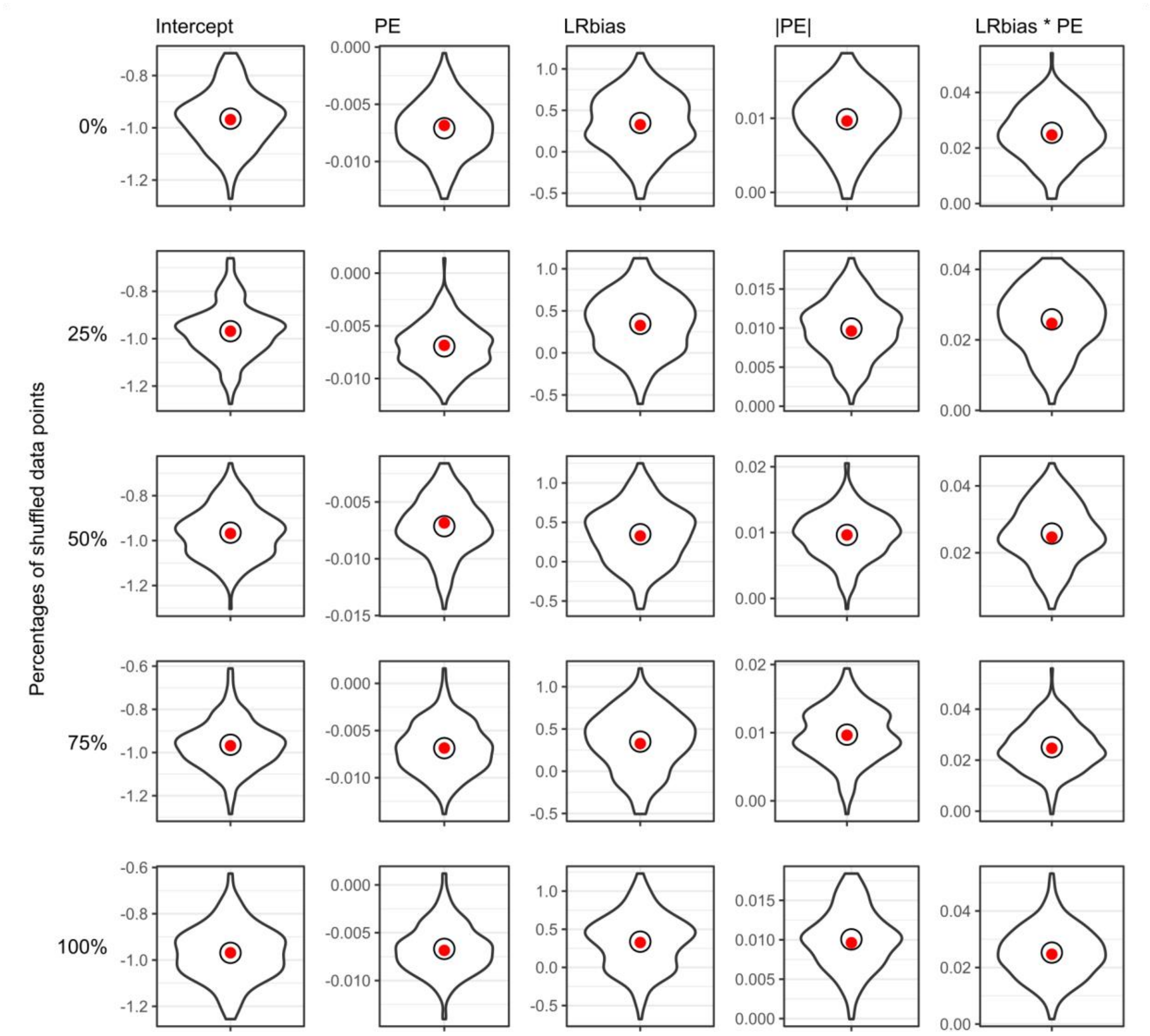

Supplementary Figure 7. Visual representation of parameter recovery for mixed model effects in the pupil data. See **Supplementary Note 6** for a detailed description. Empirical FFX estimates (as red dots) overlayed on top of violin plots of the recovered estimates; the means of the recovered parameters are displayed as empty black circles

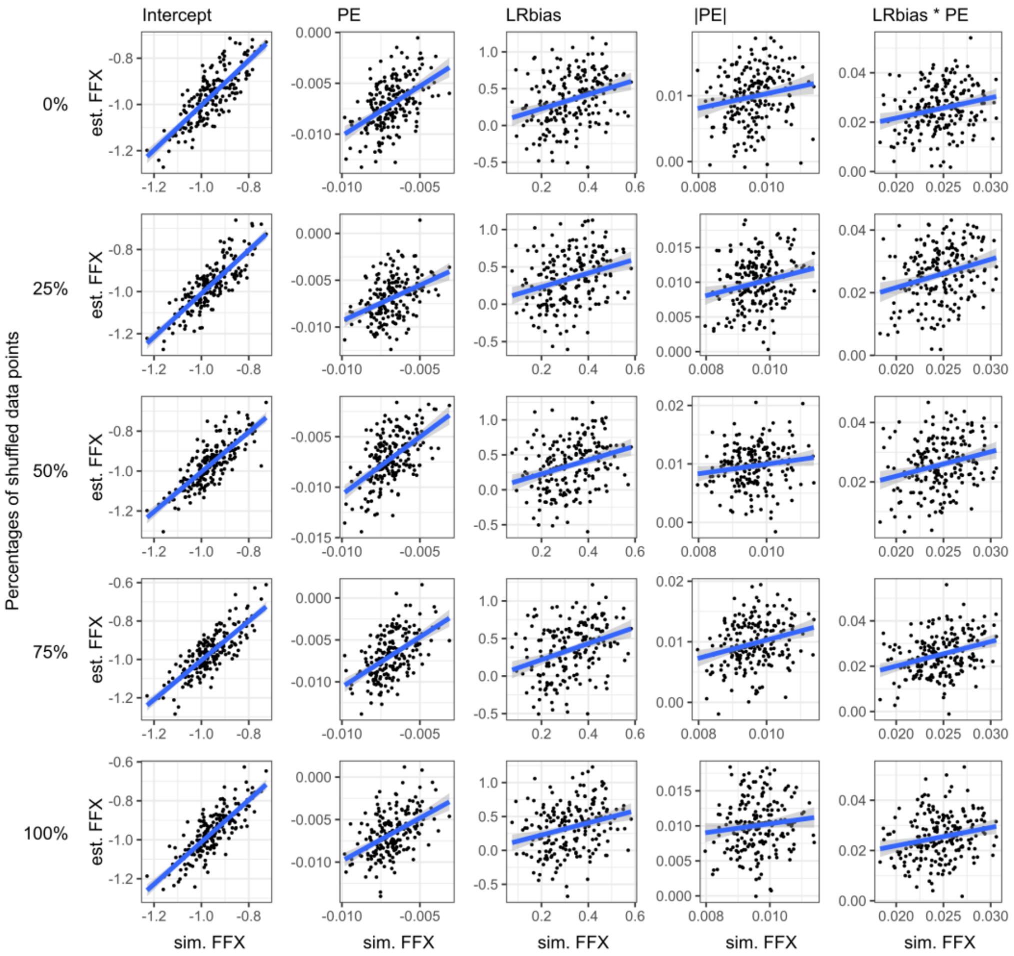

Supplementary Figure 8. Correlation plots for parameter recovery analyses. Correlation plots between each set of recovered FFX and the corresponding parameter underlying the simulated data. Blue trend lines are slopes fitted by linear regression and shadings show the 95% confidence intervals for each trend line. See **Supplementary Note 6** for a detailed description.

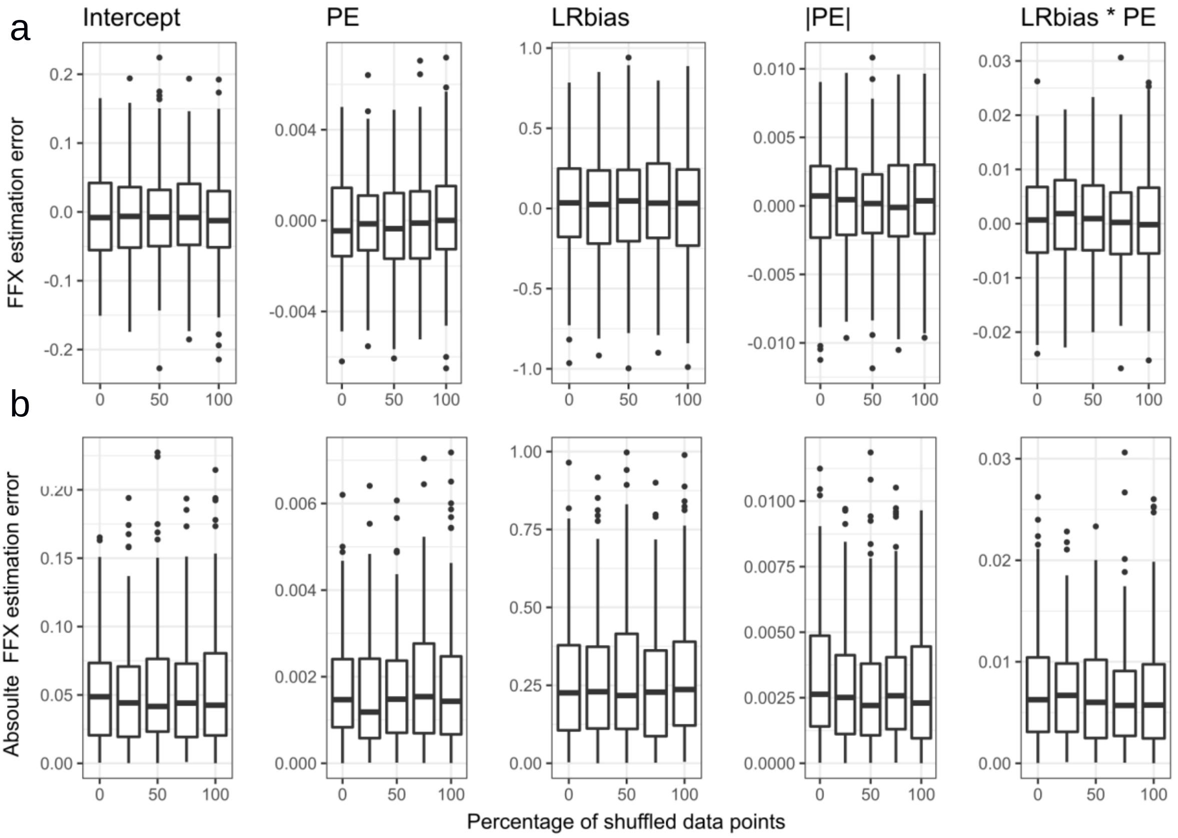

Supplementary Figure 9. Differences between recovered and underlying parameters for mixed model effects in the pupil data. a) Box plots for the differences between recovered and underlying parameters (FFX estimation error) for each fixed effect (intercept, signed PE, LRbias, unsigned PE, LRbias * signed PE) and all levels of tiling. **b)** Box plots for the absolute differences between recovered and underlying parameters (absolute FFX estimation error) for each fixed effect (intercept, signed PE, LRbias, unsigned PE, LRbias * signed PE) and all levels of tiling. Lower and upper box borders define the first and third quartile and the thick horizontal line within each box marks the median. Whiskers extend from the upper (lower) box borders to the largest (smallest) data point at most 1.5 times the interquartile range above (below) the respective border. Data with more extreme values than this are displayed as individual points. See **Supplementary Note 6** for a detailed description.

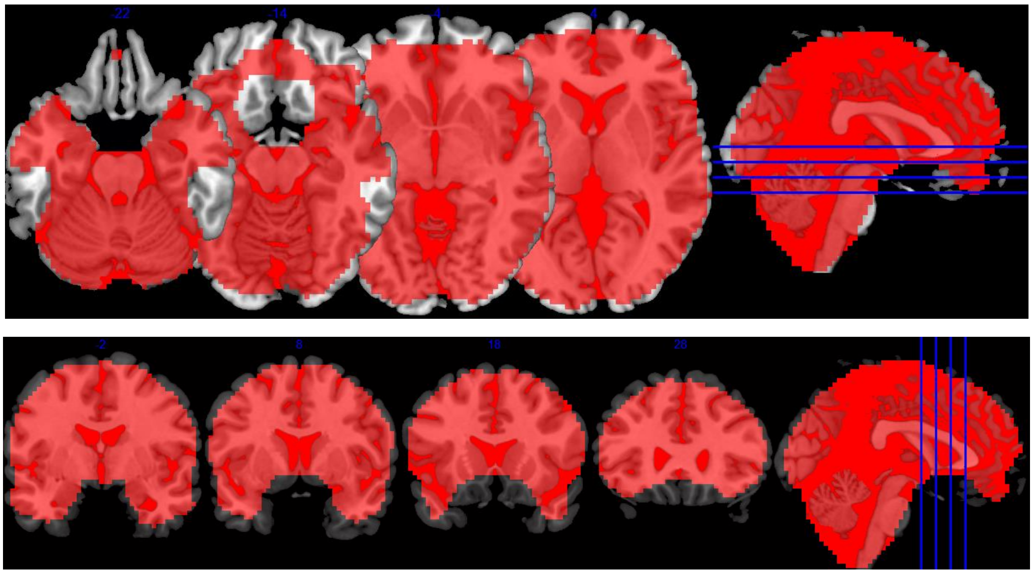

Supplementary Figure 10. Brain mask of the 2nd level fMRI analyses. Brain coverage and signal loss was as expected for fMRI studies with preserved signal in our regions of interest. Red shading shows the whole brain mask as derived from the normalized EPI images across subjects, i.e. those voxels that show sufficient signal in all included individuals. Blue lines show the origin of each of the slices depicted within the respective MNI coordinate axis (z above and y below).

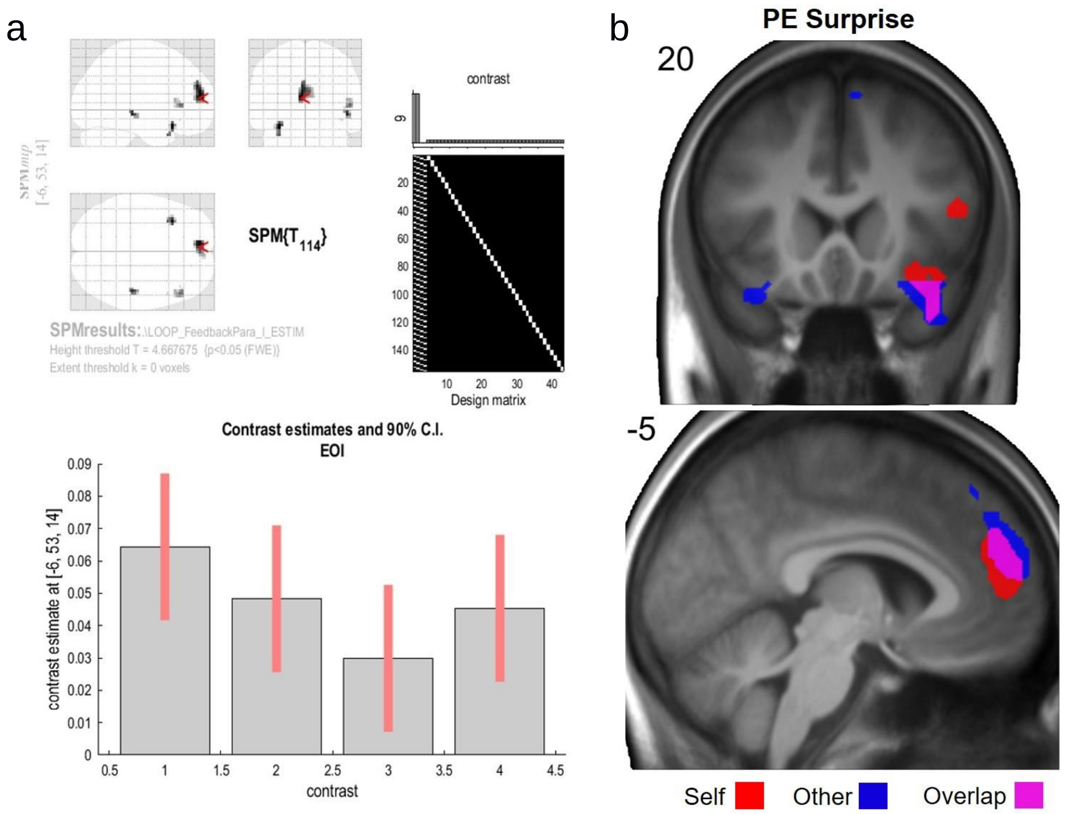

Supplementary Figure 11. Visual comparison of effects when assessing parametric weights separately or combined for positive vs negative feedback conditions: PE valance effects. a) PE valence effect for Self-Positive > Self-Negative. The flexible factorial model includes parametric weights for PEs for Self-Positive (1), Self-Negative (2), Other-Positive (3), and Other-Negative (4). The contrast PE valence for Self-Positive > Self-Negative is depicted and parameter estimates are shown for the peak voxel in the mPFC. The contrast combines two effects: The effect of PE valence for the Self-Positive contrast entails the PE valence and also the PE surprise effect. The negative PE Valence effect of Self-Negative also entails the positive PE surprise effect. As PE surprise effects add up for the two conditions (Self-Positive and Self-Negative) and PE valence effects are subtracted here, the effect shown is therefore comparable to the PE surprise effect in our original analysis as indicated by the parameter estimates for the mPFC. Results are depicted for illustration purposes and visual comparison with our original analytical approach described in the **Methods** section. The Other conditions are of no interest here. Parametric weights for PEs are coded as unsigned values in the GLM which explains the contrast weights in the Self-Negative condition (i.e. -1 codes more positive PEs). **b)** Shows the corresponding PE surprise effect in our current analyses for visual comparison (see also **Figure 4**).

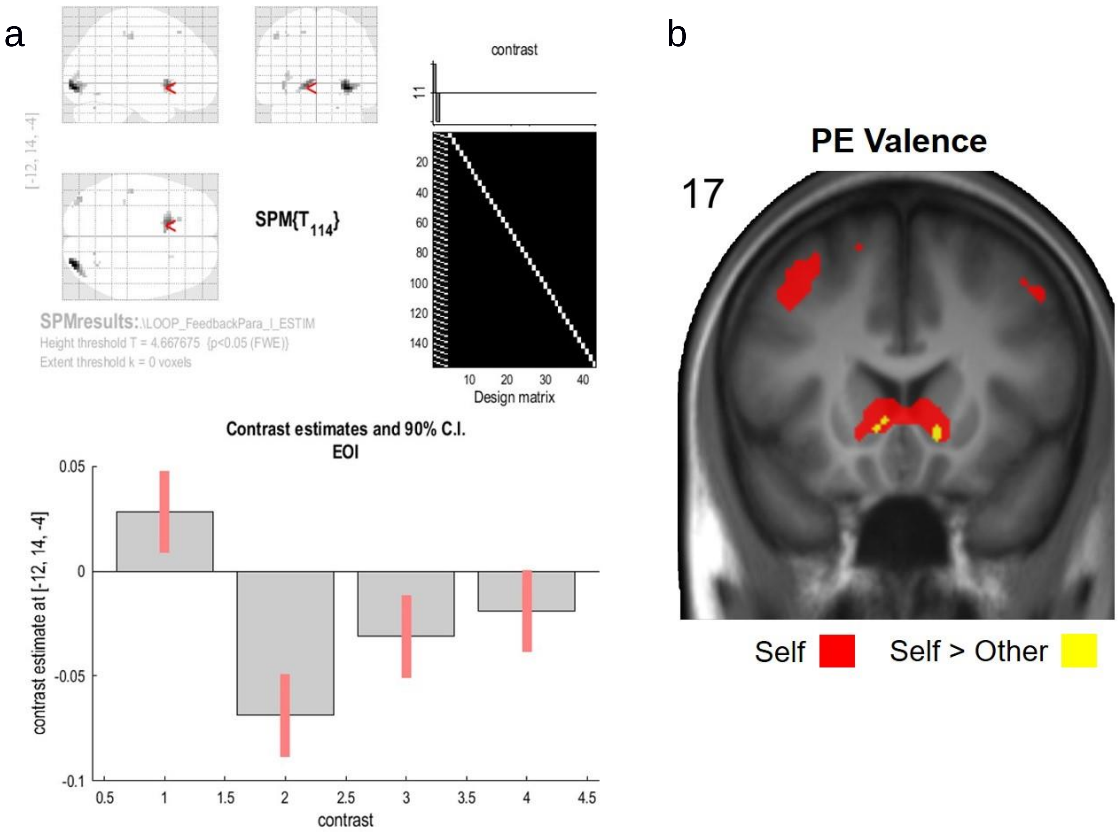

Supplementary Figure 12. Visual comparison of effects when assessing parametric weights separately or combined for positive vs negative feedback conditions: PE surprise effects. a) PE surprise for Self-Positive > Self-Negative. The flexible factorial model includes parametric weights for PEs for Self-Positive (1), Self-Negative (2), Other-Positive (3), and Other-Negative (4). The contrast PE surprise for Self-Positive > Self-Negative is depicted and parameter estimates are shown for the peak voxel in the ventral striatum. The effect of PE surprise Self-Positive again entails PE valence besides the PE surprise effect and the negative PE surprise effect of Self-Negative also entails the positive effect of PE Valence. Here, PE valence effects add up and PE surprise are subtracted. Results are therefore comparable to the PE valence effect in our original analysis as indicated by the parameter estimates for the ventral striatum. Results are depicted for illustration purposes and visual comparison with our original analytical approach described in the **Methods** section. The Other conditions are of no interest here. Parametric weights for PEs are coded as unsigned values which explains the contrast weights in the Self-Negative condition (i.e. -1 codes more positive PEs). **b)** Shows the corresponding PE valence effect in our current analyses (derived from **Figure 4**).

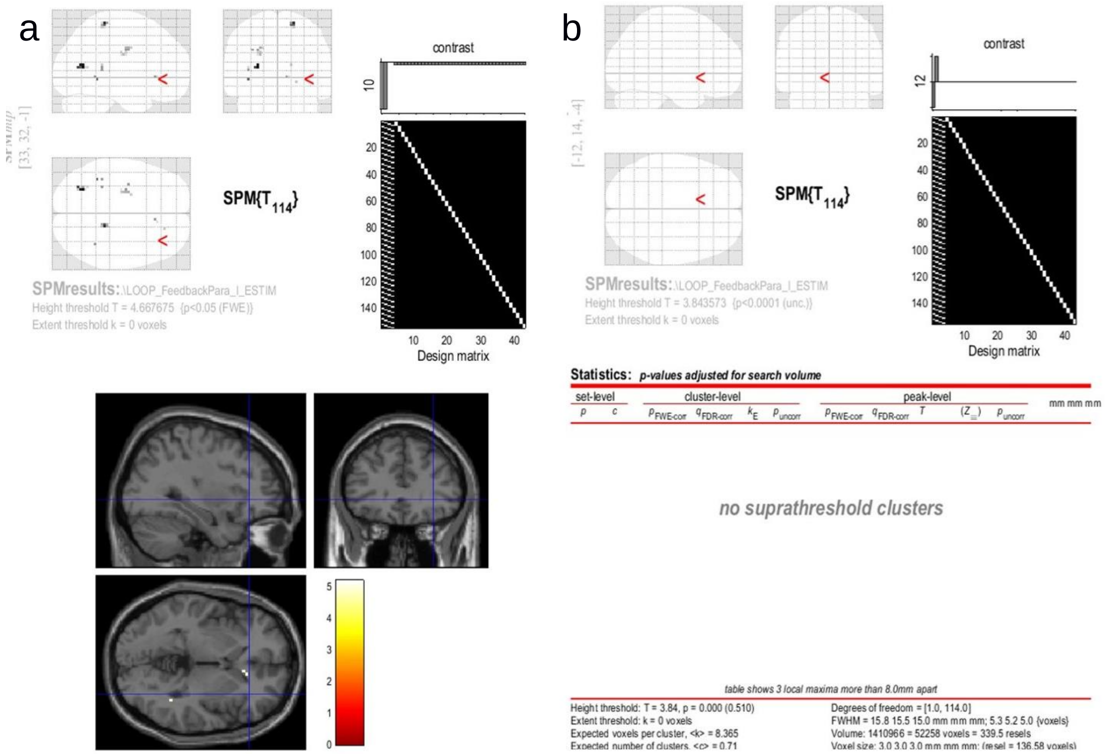

Supplementary Figure 13. Effects when assessing parametric weights separately for positive vs negative feedback conditions: Self-Negative > Self-Positive. a) PE valence for Self-Negative > Self-Positive, the opposite comparisons as shown in **Supplementary Figure 11,** did not yield any further effects **b)** PE surprise for Self-Negative > Self-Positive, the opposite comparisons as shown in **Supplementary Figure 12,** did not yield any further effects. The flexible factorial model includes parametric weights for PEs for Self-Positive (1), Self-Negative (2), Other-Positive (3), and Other-Negative (4). Results are depicted for illustration purposes and visual comparison with our original analytical approach described in the **Methods** section. The Other conditions are of no interest here. Parametric weights for PEs are coded as unsigned values which explains the contrast weights in the Self-Negative condition (i.e. -1 codes more positive PEs).

Supplementary Notes

Supplementary Note 1: Model free behavioral analyses reveal more negative self-evaluation.

First, we performed a model-free analysis to capture the basic effects observed in our behavioral data. Analyses of behavioral data and learning rates are based on the combined fMRI (n=39) and behavioral sample (n=30; total sample N=69). The Trial x Ability condition x Agent condition linear mixed model revealed a significant main effect of Ability condition (β=-20.54, $t_{(68)}$=-13.45, p<.001, *95% CI*=[-23.54; -17.55]) and interaction of Trial x Ability condition (β=-1.23, $t_{(5240)}$=-36.55, p<.001, *95% CI*=[-1.30; -1.16]), indicating that participants adapted their performance expectation ratings over time according to the feedback provided in each Ability condition (see **Figure 1c**). Moreover, there was a significant main effect of Agent condition (β=6.78, $t_{(68)}$=6.52, p<.001, *95% CI*=[4.74; 8.82]), indicating that participants evaluated their own performance more negatively than the other's performance. There was also a significant interaction of Agent condition x Ability condition (β=1.29, $t_{(5240)}$=3.33, p<.001, *95% CI*=[0.53; 2.05]). The three-way interaction of Trial x Agent condition x Ability condition (β=0.18, $t_{(5240)}$=2.66, p<.001, *95% CI*=[0.05; 0.31]) revealed a significant effect, hinting at differential learning patterns between the Ability conditions for Self vs. Other.

Supplementary Note 2: Posterior predictive checks: Behavioral analyses on the predicted data.

To assess whether our winning model captured the core effects in our model free analysis, we let the parametrized winning model predict the time course of EXP for each participant, and compared these model predictions against the actual data (see **Figure 1c**). **Figure 1c** visually confirms the ability of the model to capture the observed data despite its small number of parameters. We repeated the behavioral analyses we had done on the actual behavioral data on the predicted data. The Trial x Ability condition x Agent condition linear mixed model on the predicted data revealed a significant main effect of Ability condition (β=-19.91, $t_{(68)}$=-14.55, p<.001, *95% CI*=[-22.59; -17.22]) and interaction of Trial x Ability condition (β=-1.29, $t_{(5240)}$=-55.98, p<.001, *95% CI*=[-1.34; -1.25]) replicating the effect that participants learned over time. More negative performance expectations for the self could also be replicated as indicated by the main effect of Agent (β=6.88, $t_{(68)}$=6.50, p<.001, *95% CI*=[4.80; 8.95]). The significant interaction of Agent condition x Ability condition (β=1.12, $t_{(5240)}$=4.22, p<.001, *95% CI*=[0.60; 1.65]) indicated differential ability beliefs between the

Ability conditions for self vs other. Only the three-way interaction of Trial x Agent condition x Ability condition (β=0.04, $t_{(5240)}$=0.88, p=.377, *95% CI*=[-0.05; 0.13]) failed to reach significance. The analysis largely repeats the behavioral analysis done on the model-free data onto the predictions thus confirming that it recapitulates the main effects in our data.

Supplementary Note 3: Model parameter checks.

To assess whether introducing *w* as an additional parameter compared to our previous publications [1] we assessed correlations between the same parameters from the simple Valence Model 5 and our winning Model 8. Parameter correlations were rather high (Valence Learning Bias: ρ=.87, p<.001, $\alpha_{Self/PE+}$: ρ=.82, p<.001, $\alpha_{Self/PE-}$: ρ=.86, p<.001) and we also found a similar negative Valence Learning Bias in both models (testing both Valence Learning Biases against zero: Model 8: *mean*=-.12, t=-2.97, p=.004; Model 5: *mean*=-.12, t=-3.13 p=.003) indicating that including w does not change the results or interpretation in a meaningful way.

Supplementary Note 4: Neural activations associated with feedback processing indicate a specific role of Prediction Error Sign during self-related learning.

To examine the brain processes that underlie how people form self- and other-related ability beliefs, we compared neural activation during feedback processing as measured with fMRI. On the subject level, the fixed-effects GLM assessing effects of the different feedback conditions included four epoch regressors modeling the hemodynamic responses to the different cue conditions (Ability: High vs. Low × Agent: Self vs. Other), weighted with the performance expectation ratings per trial as parametric modulator for each condition. Four regressors modeled the four feedback conditions (Prediction Error Sign: Positive vs. Negative × Agent: Self vs. Other), each weighted with the PE value for each trial. Here, in line with the behavioral learning model, Prediction Error Sign does not correspond to Ability condition but refers to the categorical distinction between Feedback with positive PE vs negative PEs. One regressor modeled the performance expectation rating phase. The estimation periods for Self and Other were modeled as two regressors, and emotion ratings phase and the instruction phase as separate regressors. Each of the regressors was modeled with the exact duration as presented during the experiment: The cue phase was modeled with a duration of 2.5 secs, the expectation rating phase according to individual reaction times with a mean of 4.26 sec (SD=1.04), the estimation phase with 10 secs, the feedback phase with 3 secs, and the emotion rating phase with 22.51 sec (SD=3.85). To account for noise due to head movement, six additional regressors modeling head movement parameters were introduced and a constant

term was included for each of the two sessions. On the second level, beta images for the four feedback conditions were included in a flexible factorial design with two repeated measurement factors (Prediction Error Sign and Agent).

We found that the bilateral insula, anterior cingulate cortex, and thalamus (amongst others, see **Supplementary Figure 4** and **Supplementary Table 7**) were activated significantly more strongly for self-related compared to other-related performance feedback (i.e. Agent effect). This finding of heightened activity in brain regions that have been linked to arousal, but also to self-agency, potentially reflects a difference in the subjective salience of self- vs. other-related information [2–4]. Compared to feedback for the Self, feedback for the Other resulted in stronger activation of the left and right middle temporal gyrus and precuneus/ middle cingulate gyrus (**Supplementary Table 7**).

Second, we compared self-related positive vs. negative feedback in order to examine how the valence of information affected neural processing (categorical Prediction Error Sign effect). We found significantly stronger activations of the left and right nucleus accumbens/ ventral striatum (NAcc/VS), bilateral angular gyrus, medial prefrontal cortex (mPFC), and precuneus/ posterior cingulate cortex (PCC) for positive Prediction Error Sign than for negative Prediction Error Sign (see **Supplementary Table 7**). This valence effect was unique for the processing of self-related information and did not emerge for other-related performance feedback (no significant clusters for the Prediction Error Sign effect for Other; $p < .001$). The opposite contrast, negative vs. positive Prediction Error Sign, yielded no significant activations, either for self-related or for other-related information. When testing the interaction of Agent x Prediction Error Sign, we found increased activation for self-related positive vs. negative feedback ([Self positive PE > Self negative PE] > [Other positive PE > Other negative PE]) in the angular gyrus (see **Supplementary Table 7**), and at a more lenient threshold also the bilateral NAcc/VS, the precuneus/ PCC, and precentral gyrus (cluster-wise FWE-corrected with $p < .05$ at a cluster forming threshold of $p < .001$; see **Supplementary Figure 4** and **Supplementary Table 8**).

Supplementary Note 5: Specific associations of embarrassment and pride with neural activity in response to self-related PE valence.

To test whether embarrassment and pride had independent effects on neural activity in response to self-related PE valence within our predefined ROIs, we extracted parameter estimated for the effect of the parametric weights for PE valence for each whole ROI. Mean parameter estimates for the whole ROIs were then entered into regression models predicting

the neural activity with both affect ratings simultaneously. We found independent effects of pride (β=0.36, $t_{(36)}$=2.63, p=.012) and embarrassment (β=-0.39, $t_{(36)}$=-2.82, p=.008; R^2=.33, $F_{(2,36)}$=8.94, p<.001) within the amygdala. We also found independent effects of pride (β=0.43, $t_{(36)}$=3.17, p=.003) and embarrassment (β=-0.36, $t_{(36)}$=-2.63, p=.013; R^2=.36, $F_{(2,36)}$=10.10, p<.001) within the dAI. For the vAI we found a significant effect of pride (β=0.38, $t_{(36)}$=2.61, p=.013) and a trend-wise effect of embarrassment (β=-0.29, $t_{(36)}$=-2.01, p=.052; R^2=.26, $F_{(2,36)}$=6.47, p=.004). Independent effects for pride (β=0.39, $t_{(36)}$=2.85, p=.007) and embarrassment (β=-0.40, $t_{(36)}$=-2.91, p=.006; R^2=.36, $F_{(2,36)}$=9.97, p<.001) were also present for the mPFC and we also found independent effects of pride (β=0.32, $t_{(36)}$=2.39, p=.022) and embarrassment (β=-0.46, $t_{(36)}$=-3.37, p=.002; R^2=.36, $F_{(2,36)}$=10.20, p<.001) for the VTA/ SN.

Supplementary Note 6: Assessment of dependencies between PE surprise and PE valence for linear mixed model analyses

The sizes of signed PEs (PE valence) and unsigned PEs (PE surprise) always matches, i.e. there are no instances, by definition, in which a large signed PE co-occurs with a small unsigned PE, or vice versa (“tiling”). We, therefore, conducted some control analyses to assess whether the fixed effects (FFX) estimates obtained by fitting our mixed models are unaffected by this.

We simulated 200 sets of new pupil dilation data for each subject for five different degrees of tiling of signed and unsigned PEs (see below). We then obtained FFX estimates from each of these 200 sets of simulated data to test whether the distributions of FFX estimates obtained by our mixed models were affected by tiling. For both data simulation and model estimation we used the same underlying model as used to analyze the empirical data reported in the manuscript. All simulations were based on the empirical data and the distributions of effect estimates (FFX and RFX) obtained from these data. This means that the RFX intercepts and noise component in the simulated data were generated by drawing from a normal distribution with zero mean and a standard deviation equal to the RFX intercepts and residuals, respectively, from our empirical models. We only changed the data for unsigned PEs before generating a new set of simulated data: precisely, to induce different degrees of tiling, we shuffled a subset of the empirical unsigned PEs on each simulation run. The subset was randomly selected from all 1440 data points on each simulation and different percentages of these data points were shuffled (0%, 25%, 50%, 75%, or 100%). On each simulation run, we created a single set of new regression weights for each participant which was then combined with each of the five levels of tiling to compute new data based on the first-level regression

formulas. A noise component was added. Afterwards, these simulated datasets were analyzed with the same mixed model used in the manuscript, to recover FFX estimates for the parameters underlying each set of simulated data.

First, we verified that the FFX recovered from simulated data were in a domain of parameter space that, as intended, coheres with our empirical FFX (see **Supplementary Figure 7**). T-tests showed no significant differences between empirical and recovered FFX (all p>.07, uncorrected). Moreover, no differences between levels of tiling were found for directed (all ps>.154) or absolute deviations (all ps>.184) of recovered from empirical FFX. Together, this demonstrates that recovered FFX were in realistic domains of parameter space, and independent of tiling levels. Second, we tested whether the FFX used for data simulation were positively correlated with the recovered FFX. To do so, we tested correlations between each set of recovered FFX (e.g., the intercepts) and the corresponding parameter underlying the simulated data (see **Supplementary Figure 8**). Here, all correlations were positive (all ps<.032, Holm-corrected), with the only exception being the FFX for unsigned PEs in a single level of shuffling (100%; completely even tiling), which just missed significance with p=.053. In a final step, we tested whether the degree of tiling affected the quality of FFX recovery. For each fixed effect (intercept, signed PE, Valence Learning Bias, unsigned PE, Valence Learning Bias * signed PE) we tested whether levels of tiling induced over- or underestimations of FFX (as compared to the parameters underlying the simulated data) by using the differences between recovered and underlying parameters as the dependent variable. No such effect was found for any of the five FFX (all p>.153; see **Supplementary Figure 9**). In a similar fashion, we tested whether the precision of FFX recovery was affected by levels of tiling by performing equivalent analyses of variance for the absolute differences between recovered and underlying parameters. Again, no such effect was found for any parameter (all p>.194; see **Supplementary Figure 9**). Together, our analyses demonstrate that differences in tiling of signed and unsigned PEs did not induce systematic biases during data simulation or the estimation of FFX from these simulated data.

Supplementary Tables

Supplementary Table 1. PSIS-LOO Scores

Model	PSIS-LOO	LOO-SE	LOO-Diff (SE-Diff)	% of $\hat{k} > 0.7$	No. Est. Parameters
Mean Model (M0)	-2644.4	319.7	1436.1 (142.8)	0.07	4
**Self = Other**					
Unity Model (M1)	-1801.3	396.5	593.0 (109.0)	0.47	5
Context Model (M2)	-1681.2	367.8	472.9 (80.6)	0.58	6
Valence Model (M3)	-1679.3	388.0	470.9 (93.9)	0.74	6
**Self ≠ Other**					
Unity Model (M4)	-1621.2	363.6	412.9 (75.5)	0.34	6
Context Model (M5)	-1599.9	372.6	391.6 (69.2)	1.43	8
Valence Model (M6)	-1346.4	333.6	138.1 (39.0)	0.53	8
ext. Valence Model (M7)	-1251.4	349.2	43.1 (16.7)	1.58	9
ext. Valence Model (M8)	-1208.3	357.7	-	1.39	9

Note. LOO = sum PSIS-LOO, approximate leave-one-out cross-validation (LOO) using Pareto-smoothed importance sampling (PSIS); LOO-SE = Standard error of PSIS-LOO; LOO-Diff (SE-Diff) = Difference in expected predictive accuracy (PSIS-LOO) for all models from the model with the highest PSIS-LOO (extended Valence Model M8) and standard errors of differences; percentage of $\hat{k}$ - estimated shape parameters of the generalized Pareto distribution - exceeding 0.7 (all according to Vehtari et al. [5]; No. Est. Parameters = number of estimated parameters in the model.

Supplementary Table 2. Initial and final ability beliefs during the LOOP task.

		Initial Belief		Final Belief	
		Mean	SD	Mean	SD
Self					
	High Ability	51.4	8.3	61.1	15.7
	Low Ability	53.3	8.3	30.7	13.8
Other					
	High Ability	57.1	6.3	69.9	9.3
	Low Ability	58.9	5.9	39.7	14.1

Note. Mean performance expectation ratings for the first trial (inital belief) and for the last trial of the experiment (final belief) for each of the four Ability conditions. SD = standard deviation.

Model Parameters	SV1	SV2	SV3	SV4	$\alpha Self_{/PE+}$	$\alpha Self_{/PE-}$	$\alpha Other_{/PE+}$	$\alpha Other_{/PE-}$	BiasSelf	BiasOther
SV1		0.58*	0.33*	0.13	0.34*	-0.34*	0.06	0.1	0.46*	-0.01
SV2	0.58*		0.11	0.30*	0.12	-0.19	-0.16	-0.24*	0.23	0.16
SV3	0.33*	0.11		0.21	0.23	0.03	0.28*	0.32*	0.07	-0.11
SV4	0.13	0.30*	0.21		0.12	-0.27*	-0.18	-0.31*	0.40*	0.14
$\alpha Self_{/PE+}$	0.34*	0.12	0.23	0.12		0.11	0.45*	0.43*	0.52*	0.04
$\alpha Self_{/PE-}$	-0.34*	-0.19	0.03	-0.27*	0.11		0.17	0.19	-0.70*	-0.08
$\alpha Other_{/PE+}$	0.06	-0.16	0.28*	-0.18	0.45*	0.17		0.80*	0.06	0.14
$\alpha Other_{/PE-}$	0.10	-0.24*	0.32*	-0.31*	0.43*	0.19	0.80*		0.02	-0.40*
BiasSelf	0.46*	0.23	0.07	0.40*	0.52*	-0.70*	0.06	0.02		0.113
BiasOther	-0.01	0.16	-0.11	0.14	0.04	-0.08	0.14	-0.40*	0.11	

Supplementary Table 3. Pearson correlations for all model parameters of the winning Valence Model 8 as well as the Valence Learning Bias for Self and Other calculated from the learning rates as described in the methods section. * $p<.05$.

	Spearman Correlations				
	Valence Learning Bias	Embarrassment	Pride	Happiness	Tension
Valence Learning Bias		-0.24*	0.55**	0.23	-0.08
Embarrassment	-0.24*		-0.10	-0.07	0.53**
Pride	0.55**	-0.10		0.39**	0.20
Happiness	0.23	-0.07	0.39**		-0.07
Tension	-0.08	0.53**	0.20	-0.07	

Supplementary Table 4. Spearman correlations for all emotion ratings and the Valence Learning Bias. * p<.05; ** p<.01.

Supplementary Table 5. Activations Associated with PE Surprise

Contrasts/ Brain regions	Side	Cluster Size	MNI Coordinates			*T*	*p*
			x	y	z		
**Self: PE Surprise**							
Paracingulate Gyrus/ Superior Frontal Gyrus	R/L	12	-6	50	20	5.75	.009
Temporal Pole/ Frontal Orbital Cortex	L	8	-39	17	-22	5.57	.014
Superior Frontal Gyrus	R	1	12	20	62	5.20	.037
Temporal Pole/ Frontal Orbital Cortex	L	1	-30	11	-28	5.10	.048
**Other: PE Surprise**							
Temporal Pole/ Frontal Orbital Cortex	L	16	-33	17	-28	6.90	.001
Temporal Pole/ Frontal Orbital Cortex	R	25	39	20	-28	6.75	.001
Angular Gyrus/ Posterior Supramarginal Gyrus	R	23	48	-46	26	6.48	.002
Superior Frontal Gyrus/ Frontal Pole	R/L	74	6	53	26	6.41	.002
			-3	53	23	6.24	.003
Frontal Pole/ Superior Frontal Gyrus	R	3	9	47	47	5.50	.023
Posterior Supramarginal Gyrus/ Angular Gyrus	L	1	-51	-49	17	5.35	.033
Temporal Pole	R	1	48	17	-31	5.27	.042

Note. PE surprise refers to the unsigned prediction error values as parametric modulator for the feedback phase. The *p*-values are FWE corrected at peak level for the whole brain.

Supplementary Table 6. Activations Associated with PE Valence

Contrasts/ Brain Regions	Side	Cluster Size	MNI Coordinates			*T*	*p*
			x	y	z		
**Self: PE Valence**							
Superior/ Middle Frontal Gyrus	L	197	-15	29	53	7.07	<.001
Middle/ Superior Frontal Gyrus			-36	17	50	6.38	.002
Middle Frontal Gyrus			-39	23	38	5.75	.009
Superior Parietal Lobule/Superior Lateral Occipital Cortex	L	199	-36	-58	56	6.96	<.001
Angular Gyrus/ Posterior Supramarginal Gyrus			-45	-55	35	6.77	.001
Caudate / Accumbens	L	139	-9	20	-1	6.76	.001
Caudate / Accumbens	R		12	17	-1	6.41	.002
Superior Lateral Occipital Gyrus/ Angular Gyrus	R	121	48	-61	41	6.48	.001
Posterior Supramarginal Gyrus/ Angular Gyrus			51	-46	47	6.34	.002
Superior Parietal Lobule/ Angular Gyrus			39	-55	56	5.44	.020
Postcentral Gyrus/ Superior Parietal Lobule	L	50	-45	-34	56	6.08	.004
Postcentral Gyrus/ Posterior Supragarginal Gyrus			-45	-28	41	5.53	.016
**Self > Other: PE Valence**							
Accumbens	L	19	-9	26	-1	5.77	0.008
Caudate/ Accumbens	R	2	12	17	-4	5.23	0.034

Note. PE valence refers to the signed prediction error values as parametric modulator for the feedback phase. The *p*-values are FWE corrected for the whole brain at peak level. Only clusters with more than 50 voxels are reported for the Self: PE Valence contrast.

Supplementary Table 7. Activations Associated with Feedback Processing: Interaction of Agent * Prediction Error Sign

Contrasts/ Brain Regions	Side	Cluster Size	MNI Coordinates			*T*	*p*
			x	y	z		
**Interaction: Self > Other, Positive > Negative**							
Angular Gyrus/ Superior Lateral Occipital Cortex	R	229	48	-58	41	5.28	.002
			57	-61	20	3.55	
Angular Gyrus / Superior Parietal Lobule	L	303	-42	-55	44	4.78	.001
			-42	-55	53	4.76	
Angular Gurus / Posterior Supramarginal Gyrus			-48	-55	29	4.57	
Putamen/ Pallidum	R	380	18	5	-10	4.67	<.001
Caudate / Accumbens	R		12	20	-1	4.56	
Caudate / Accumbens	L		-9	20	-1	4.55	
Precentral gyrus	L	162	-18	-19	53	3.95	.008
			-27	-13	44	3.79	
			-24	-25	41	3.71	
Posterior Cingulate Gyrus/ Precuneous Cortex	R/L	296	-15	-43	32	3.91	.001
Precuneous Cortex/ Posterior Cingulate Gyrus			-3	-58	35	3.87	
Posterior Cingulate Gyrus/ Precuneous Cortex			6	-43	26	3.73	
Cerebellum Left Crus I / Crus II	L	154	-12	-82	-25	3.73	.009
Occipital Fusiform Gyrus / Cerebellum Left Crus I			-30	-79	-1	3.69	
Occipital Fusiform Gyrus / Inferior Lateral Occipital Cortex			-30	-85	-10	3.65	

Note. Cluster extents refer to $p < .001$, uncorrected and p-values are FWE corrected on the cluster level.

Supplementary Table 8. Differential Functional Connectivity of the Dorsal Anterior Insula Associated with PE Valence

Covariates/ Regions of interest	Side	Cluster Size	MNI Coordinates			*T*	*p*
			x	y	z		
**PPI Right Dorsal Anterior Insula**							
Amygdala	R	6	33	-1	-31	4.46	.003
	L	2	-27	-4	-25	3.89	.013
		5	-24	2	-13	3.68	.022
Ventral Tegmental Area/ Substantia Nigra	R/L	5	-18	-16	-13	4.07	.015
Medial Prefrontal Cortex	R/L	11	-9	35	53	4.95	.005
		5	-6	59	26	4.62	.012
**PPI Left Dorsal Anterior Insula**							
Amygdala	L	3	-30	-4	-22	3.76	.019
Ventral Tegmental Area/ Substantia Nigra	R/L	3	-9	-13	-13	3.66	.042

Note. Stronger functional connectvity for negative vs positive PEs for self- vs other-related feedback. The *p*-values are FWE corrected within ROIs at peak level.

Supplementary Table 9. Sample Characteristics

	fMRI Sample		Behavioral Sample		p
	Mean	SD	Mean	SD	
Age	22.30	2.65	23.30	3.97	.234
Self-esteem	6.24	0.84	6.07	1.26	.522

Note. Sample characteristics for both samples. SD = standard devation; fMRI Sample: n=39, Behavioral Sample: n = 30; p-value refers to a two sample t-test, df = 67.

Supplementary References

1. Müller-Pinzler, L. *et al.* Negativity-bias in forming beliefs about own abilities. *Sci. Rep.* **9**, (2019).
2. Späti, J. *et al.* Functional lateralization of the anterior insula during feedback processing. *Human Brain Mapping* **35**, 4428–4439 (2014).
3. Sperduti, M., Delaveau, P., Fossati, P. & Nadel, J. Different brain structures related to self- and external-agency attribution: A brief review and meta-analysis. *Brain Structure and Function* **216**, 151–157 (2011).
4. Craig, A. D. B. How do you feel — now? The anterior insula and human awareness. *Nature Reviews Neuroscience* **10**, 59–70 (2009).
5. Vehtari, A., Mononen, T., Tolvanen, V., Sivula, T. & Winther, O. Bayesian leave-one-out cross-validation approximations for Gaussian latent variable models. *The Journal of Machine Learning Research* **17**, 3581–3618 (2016).
