# Supplementary material for: Neurocomputational mechanisms of affected beliefs
Source: Commun Biol. 2022 Nov 14;5:1241. doi: 10.1038/s42003-022-04165-3 (PMC9663730; doi:10.1038/s42003-022-04165-3)
Supplement: Supplementary file 3 — Description of Additional Supplementary Files [file 42003_2022_4165_MOESM3_ESM.pdf]

Description of Additional Supplementary Files

File name: Supplementary Data 1

Description: Table showing the Activations Associated with Feedback Processing.

File name: Supplementary Data 2

Description: Table showing the Variables Associated with Individual Differences in PE Valence Tracking.

File name: Supplementary Data 3

Description: The source data behind the graphs in the paper.
