# Supplementary material for: Neurocomputational mechanisms of affected beliefs
Source: Commun Biol. 2022 Nov 14;5:1241. doi: 10.1038/s42003-022-04165-3 (PMC9663730; doi:10.1038/s42003-022-04165-3)
Supplement: Supplementary file 7 — Reporting Summary [file 42003_2022_4165_MOESM7_ESM.pdf]

nature portfolio

Corresponding author(s): Laura Müller-Pinzler

Last updated by author(s): Oct 17, 2022

# Reporting Summary

Nature Portfolio wishes to improve the reproducibility of the work that we publish. This form provides structure for consistency and transparency in reporting. For further information on Nature Portfolio policies, see our Editorial Policies and the Editorial Policy Checklist.

## Statistics

For all statistical analyses, confirm that the following items are present in the figure legend, table legend, main text, or Methods section.

| n/a | Confirmed | |
|---|---|---|
| ☐ | ☒ | The exact sample size (*n*) for each experimental group/condition, given as a discrete number and unit of measurement |
| ☐ | ☒ | A statement on whether measurements were taken from distinct samples or whether the same sample was measured repeatedly |
| ☐ | ☒ | The statistical test(s) used AND whether they are one- or two-sided<br>*Only common tests should be described solely by name; describe more complex techniques in the Methods section.* |
| ☐ | ☒ | A description of all covariates tested |
| ☐ | ☒ | A description of any assumptions or corrections, such as tests of normality and adjustment for multiple comparisons |
| ☐ | ☒ | A full description of the statistical parameters including central tendency (e.g. means) or other basic estimates (e.g. regression coefficient) AND variation (e.g. standard deviation) or associated estimates of uncertainty (e.g. confidence intervals) |
| ☐ | ☒ | For null hypothesis testing, the test statistic (e.g. $F$, $t$, $r$) with confidence intervals, effect sizes, degrees of freedom and $P$ value noted<br>*Give P values as exact values whenever suitable.* |
| ☐ | ☒ | For Bayesian analysis, information on the choice of priors and Markov chain Monte Carlo settings |
| ☒ | ☐ | For hierarchical and complex designs, identification of the appropriate level for tests and full reporting of outcomes |
| ☐ | ☒ | Estimates of effect sizes (e.g. Cohen's *d*, Pearson's *r*), indicating how they were calculated |

*Our web collection on statistics for biologists contains articles on many of the points above.*

## Software and code

Policy information about availability of computer code

| | |
|---|---|
| Data collection | MATLAB Release 2015b, MATLAB Release 2019a, Psychtoolbox 3, Sosci Survey 3.2.46, |
| Data analysis | MATLAB Release 2019a, SPM12, R package version 2.14.1., Rstan, jamovi version 1.2.27 |

For manuscripts utilizing custom algorithms or software that are central to the research but not yet described in published literature, software must be made available to editors and reviewers. We strongly encourage code deposition in a community repository (e.g. GitHub). See the Nature Portfolio guidelines for submitting code & software for further information.

## Data

Policy information about availability of data

All manuscripts must include a data availability statement. This statement should provide the following information, where applicable:

- Accession codes, unique identifiers, or web links for publicly available datasets
- A description of any restrictions on data availability
- For clinical datasets or third party data, please ensure that the statement adheres to our policy

The data that support the findings of this study are available from the corresponding author upon reasonable request.

# Field-specific reporting

Please select the one below that is the best fit for your research. If you are not sure, read the appropriate sections before making your selection.

☐ Life sciences ☒ Behavioural & social sciences ☐ Ecological, evolutionary & environmental sciences

For a reference copy of the document with all sections, see nature.com/documents/nr-reporting-summary-flat.pdf

# Behavioural & social sciences study design

All studies must disclose on these points even when the disclosure is negative.

| | |
|---|---|
| Study description | Experimental, within-subjects study, between-subject covariates, with quantitative data. |
| Research sample | Convenience sample mostly comprised of undergraduate and graduate students from Lübeck University (Germany). Subjects were excluded in case they had pre-existing psychiatric or neurological conditions. fMRI sample: aged 18-28 years; M=22.3; SD=2.65; behavioral sample: aged 18-32 years; M=23.3; SD=3.97 |
| Sampling strategy | Convenience samples were recruited using university mailing lists and leaflets spread on the campus of Lübeck University. Participants received monetary compensation or, if requested, partial course credit. Prior to this study we had little evidence to rely on for estimating the effect sizes but expected medium to large effects for fMRI effects within-subject manipulations and targeted a sample of N = 45 to achieve sufficient power for the three studies, including roughly 10% exclusions due to subjects not believing the coverstory. The final fMRI sample consisted of 39 subjects. |
| Data collection | For the behavioral sample, data were collected using a desktop computer that was placed in an isolated room in which subjects were seated. Research staff left the room after the subjects finished reading the task instructions, and subjects remained seated until the end of the experimental paradigm. In the fMRI study, subjects performed the experiment via an MRI-compatible screen placed behind the scanner bore and visible to the subjects via a mirror mounted to the headcoil. Eytracking was assessed via an Eyelink eytracker system. In the fMRI study, research staff was present at all times in the MRI control room, in order to monitor data acquisition. A confederate or second participant was present in the beginning of the study, before participants (or confederate) were lead into separate rooms. Subjects completed forms regarding demographic data and questions about the experimental paradigms using Sosci Survey. Research staff performing data collection was not blind to the experimental conditions but conditions were manipualted within subjects. |
| Timing | Data collection: start March 2019 until approximately Dezember 2019 |
| Data exclusions | Exclusion of 6 participants who did not believe the cover story of the task and 3 participants who did not attentively complete the task until the end. Additional exclusion of 3 participants for eytracking analyses due to insufficient data quality. |
| Non-participation | No participants dropped out or declined to participate. |
| Randomization | Participants were not allocated to experimental groups. |

# Reporting for specific materials, systems and methods

We require information from authors about some types of materials, experimental systems and methods used in many studies. Here, indicate whether each material, system or method listed is relevant to your study. If you are not sure if a list item applies to your research, read the appropriate section before selecting a response.

## Materials & experimental systems

| n/a | Involved in the study | |
|---|---|---|
| ☒ | ☐ | Antibodies |
| ☒ | ☐ | Eukaryotic cell lines |
| ☒ | ☐ | Palaeontology and archaeology |
| ☒ | ☐ | Animals and other organisms |
| ☐ | ☒ | Human research participants |
| ☒ | ☐ | Clinical data |
| ☒ | ☐ | Dual use research of concern |

## Methods

| n/a | Involved in the study | |
|---|---|---|
| ☒ | ☐ | ChIP-seq |
| ☒ | ☐ | Flow cytometry |
| ☐ | ☒ | MRI-based neuroimaging |

## Human research participants

Policy information about studies involving human research participants

| | |
|---|---|
| Population characteristics | see above |

| | |
|---|---|
| Recruitment | Subjects were recruited using student mailing lists from Lübeck University and leaflets handed out on campus. Subjects responding to these measures might show lower levels of anxiety or related phenomena. Furthermore, no subjects with preexisting psychiatric or neurological conditions were included. These factors might reduce variability in the sample with regard to affective dimensions, but should not impact the general validity of the psychological processes investigated in this manuscript. |
| Ethics oversight | Ethics committee of Lübeck University, Germany (AZ 18-066) |

Note that full information on the approval of the study protocol must also be provided in the manuscript.

# Magnetic resonance imaging

## Experimental design

| | |
|---|---|
| Design type | event-related fMRI |
| Design specifications | Two sessions were scanned with each 40 trials, overall 80 trials. Each trial was on averge approximately 28 seconds long, the intertrial interval was on average approximately 6 seconds (pseudo-jittered interval). |
| Behavioral performance measures | During the expectation rating phase we collected ratings and response times, during the estimation phase we collected response times for each subject as well as number of mouse clicks and responses chosen. Expectation ratings were self-paced and mandatory for the task to continue. During estimations we assessed if participants missed to respond and participants were excluded when they showed excessive numbers of missings (did not complete the task attentively, see sample description; mean missings without outliers = 2.84 trials, sd = 4.00). |

## Acquisition

| | |
|---|---|
| Imaging type(s) | funtional and structural (T1) for normalization |
| Field strength | 3 T |
| Sequence & imaging parameters | 60 near-axial slices, echo planar imaging (EPI) sequence, TR=0.992s, TE=28ms, flip angle=60°, voxel size=3×3×3mm 3, simultaneous multi-slice factor 4 |
| Area of acquisition | whole brain scans |

Diffusion MRI ☐ Used ☒ Not used

## Preprocessing

| | |
|---|---|
| Preprocessing software | FMRI data were analyzed using SPM12 (www.fil.ion.ucl.ac.uk/spm). Field maps were reconstructed to obtain voxel displacement maps (VDMs). EPIs were corrected for timing differences of the slice acquisition, motion-corrected and unwarped using the corresponding VDMs to correct for geometric distortions. Data were smoothed with an 8 mm full-width-at-half-maximum isotropic Gaussian kernel. |
| Normalization | For normalization, each subject's structural T1 image was coregistered to the subject's mean functional image. Using the unified segmentation approach implemented in SPM12 forward deformation fields were obtained that were then used to normalize the functional images to MNI space. |
| Normalization template | For the unified segmentation templates in MNI152 space included in SPM12 were used. |
| Noise and artifact removal | To remove low-frequency drifts, functional images were high-pass filtered at 1/384. Motion regressors derived from realignment were included as covariates in the first-level GLM analyses to account for movement-related artifacts. |
| Volume censoring | none |

## Statistical modeling & inference

| | |
|---|---|
| Model type and settings | We used a mass-univariate, first-order autoregressive GLM analysis on the first level. Contrast images from the first level were than analysed on the second-level. |
| Effect(s) tested | On the second level t-tests were used to assess baseline contrasts and repeated ANOVA models to assess differences between two with-subject conditions (e.g. Self vs Other, Negative vs Positive). All covariates were tested in simple t-tests that included a covariate as implemented in SPM12. |

Specify type of analysis: ☐ Whole brain ☐ ROI-based ☒ Both

| | |
|---|---|
| Anatomical location(s) | A bilateral ventral and a bilateral dorsal AI ROI was defined according to the three-cluster solution of Kelly and colleagues (86). The bilateral amygdala and the mPFC (label: bilateral frontal superior medial) ROIs were derived from the AAL atlas definition in the WFU PickAtlas (87). The VTA/ SN ROI described in Murty at al. (89, 90) was included. (citations see manuscript) |

| | |
|---|---|
| Statistic type for inference (See Eklund et al. 2016) | mainly voxel-wise statistcs were used, for cluster-wise statistics a cluster forming threshold of $p < .001$ was used |
| Correction | FWE-correction as implemented in SPM12 |

## Models & analysis

| n/a | Involved in the study |
|---|---|
| ☐ | ☒ Functional and/or effective connectivity |
| ☒ | ☐ Graph analysis |
| ☒ | ☐ Multivariate modeling or predictive analysis |

| | |
|---|---|
| Functional and/or effective connectivity | Seed-based psychophysiological interaction analyses were used, regression coefficient |
